# Effectiveness of Ultrasound-Guided Lavage for Rotator Cuff Calcific Tendinopathy: A Case Series Study from a Clinical and Radiological Perspective

**DOI:** 10.3390/jcm14155376

**Published:** 2025-07-30

**Authors:** Lucrezia Moggio, Michele Mercurio, Nicola Marotta, Umile Giuseppe Longo, Giorgio Gasparini, Antonio Ammendolia, Alessandro de Sire

**Affiliations:** 1 Rehabilitation Unit, Ospedale degli Infermi, 13875 Biella, Italy; lucrezia.moggio@gmail.com; 2 Physical Medicine and Rehabilitation, Department of Medical and Surgical Sciences, University of Catanzaro “Magna Graecia”, 88100 Catanzaro, Italy; nicola.marotta@unicz.it (N.M.); ammendolia@unicz.it (A.A.); alessandro.desire@unicz.it (A.d.S.); 3 Department of Orthopaedic and Trauma Surgery, University of Catanzaro “Magna Graecia”, “Renato Dulbecco” University Hospital, 88100 Catanzaro, Italy; gasparini@unicz.it; 4 Research Center on Musculoskeletal Health, MusculoSkeletalHealth@UMG, University of Catanzaro “Magna Graecia”, 88100 Catanzaro, Italy; 5 Fondazione Policlinico Universitario Campus Bio-Medico, Via Alvaro del Portillo 200, 00128 Roma, Italy; g.longo@policlinicocampus.it; 6 Research Unit of Orthopaedic and Trauma Surgery, Department of Medicine and Surgery, Università Campus Bio-Medico di Roma, Via Alvaro del Portillo 21, 00128 Roma, Italy

**Keywords:** rotator cuff calcific tendinopathy, shoulder pain, ultrasound-guided lavage, needle aspiration

## Abstract

**Background/Objectives**: Rotator cuff calcific tendinopathy (RCCT) is one of the most common causes of non-traumatic shoulder pain. To date, there is still no consensus regarding the most effective method for its treatment. Ultrasound-guided percutaneous aspiration is suggested during the reabsorption phase of calcific metaplasia. We aimed to evaluate the effectiveness of ultrasound-guided lavage for RCCT from a clinical and radiological perspective. **Methods**: We involved patients affected by RCCT of the supraspinatus tendon. The approach used for the calcification lavage was the one-needle technique, consisting in inserting a 16–18 G needle on a 20 mL syringe with 0.9% saline solution, in the calcific metaplasia, under ultrasound guidance, using an in-plane approach; the repetitive action of pressing and releasing the plunger was repeated until the contents of the syringe became milky, at which point the syringe was replaced with a new one, always containing saline solution. The physiotherapy treatment began 7 days after the procedure. We assessed the Numeric Rating Scale, the Gartner classification, the Disability of the Arm, Shoulder and Hand scale, the Constant–Murley shoulder score, and the passive range of motion of flexion and abduction. **Results**: We included 23 subjects. The analysis of the data at baseline and t1 showed a statistically significant improvement in all the functional variables (*p* < 0.05). This result was mainly evident for pain, with a *p*-value of 0.001. **Conclusions**: The findings of the present prospective case series study showed an improvement in the clinical and radiological outcomes after ultrasound-guided percutaneous aspiration for rotator cuff calcific tendinopathy.

## 1. Introduction

Rotator cuff calcific tendinopathy (RCCT) is one of the most common causes of non-traumatic shoulder pain [1]. This condition is characterised by hydroxyapatite crystals deposited in the tendons of the rotator cuff. It could affect between 2.7% and 22% of the general population, with 7.8% of subjects asymptomatic. It most commonly affects women aged between 30 and 50 years, with bilateral involvement in 10% of patients. It does not appear to be related to physical and/or work activity. The deposits are usually located in the supraspinatus (about 1.5–2 cm from the insertion) and infraspinatus tendons, traditionally identified on standard radiographs [2]. Calcium deposits may cause chronic pain, leading to impairments in daily living and professional activities [3]. The processes underlying RCCT’s development are still being debated. Calcific tendinopathy, a multifocal and cell-mediated disease, results from a metaplastic transformation of tenocytes into chondrocytes, followed by calcification inside the tendon and remodelling by phagocytosis of the metaplastic areas by multinucleated giant cells [4]. Some dysmetabolic conditions may also lead to RCCT. Harvie et al. [5] identified endocrine involvement in 64.7% of cases, while Mavrikakis et al. [6] reported an incidence of RCCT in 31.8% of diabetic subjects, compared to 10.3% of the control group. Regardless of its pathogenesis, the natural history of calcific tendinopathy can be divided into three stages, as proposed by Uhthoff [7]: The tendon transformation into fibrocartilaginous tissue characterises the pre-calcific phase. The calcific stage is divided into formative and reabsorption phases. The first one consists of the tendon deposition of calcium crystals; the second one begins after a phase of silent course of the disease—a variable period of time—when the vascular network develops in the affected area, with the subsequent phagocytosis of the calcium deposit by macrophages. In this last phase, oedema and increased intra-tendinous pressure are observed, with the possible extravasation of calcium crystals in the subacromial bursa, usually associated with the development of acute pain, which is unresponsive to common analgesics [8].

RCCT’s clinical presentation is variable and depends on both the stage of the disease and the anatomical location [9].

Conventional radiology is often the first diagnostic approach performed on patients complaining of shoulder pain when RCCT is suspected. The Gartner and Heyer classification is the most common radiographic classification applied in clinical practice, as it correlates with the histological stage, as follows: (I) well-circumscribed and dense calcification; (II) soft/dense or transparent contour; and (III) translucent and cloudy appearance without clear circumscription [10]. The deposits are visible during the calcific stages (I and II), while in the resorption phase (III) they are difficult to identify in conventional radiography. Ultrasound is a good diagnostic tool for detecting and localising calcifications within the rotator cuff tendons, with a sensitivity of 98% and a specificity of 94% [11].

RCCT treatment includes the non-invasive approaches of physical and/or work activity modification, anti-inflammatory drugs, and physical treatment, and minimally invasive treatments, including infiltration and focal extracorporeal shockwave therapy (ESWT). In this context, ultrasound-guided lavage is considered a minimally invasive percutaneous procedure to treat rotator cuff calcific tendinopathy [12]. It is only in refractory cases that operative debridement and arthroscopic tendon repair can be considered [13].

Ultrasound-guided percutaneous aspiration is indicated during the reabsorption phase of calcific metaplasia; an ultrasound pattern of well-demarcated homogeneous hyperechogenic calcification with weak posterior acoustic shadow indicates a deposition with a “soft consistency” (Gartner and Heyer type II), which is more responsive to this procedure [8]. This approach should not be performed when patients are asymptomatic, the calcification is very small (≤5 mm), or when it has migrated into the bursal space [14].

To date, there is still no consensus in the scientific literature regarding the most effective method for treating RCCT; thus, in this scenario, the purpose of the present prospective case series study was to evaluate the effectiveness of ultrasound-guided lavage for rotator cuff calcific tendinopathy, hypothesising its relevant role from a clinical and radiological perspective.

## 2. Materials and Methods

### 2.1. Participants

This prospective case series study was conducted in accordance with the Strengthening the Reporting of Observational Studies in Epidemiology (STROBE) guidelines. The participants were recruited from individuals affected by RCCT who attended physical and rehabilitative medicine and orthopaedic services from November 2024 to April 2025, during which they underwent ultrasound-guided percutaneous aspiration and rehabilitation treatment. We included subjects meeting the inclusion criteria reported above through a consecutive sampling method.

The Ethical Committee of the Campus Bio-Medico University of Rome approved the study protocol (number 78/18). The study was performed in accordance with the ethical standards of the responsible committee on human experimentation (institutional and national) and with the Helsinki Declaration of 1975, as revised in 1983. All participants were asked to carefully read and sign an informed consent form.

The patients involved in the study were affected by RCCT of the supraspinatus tendon, as documented by ultrasound, radiographic, and MRI examination [15]. The inclusion criteria were as follows: (1) patients who were aged more than 18 years at the time of the procedure; (2) supraspinatus tendon as the site of calcification; (3) calcifications larger than 0.8 cm; (4) symptomatic calcifications; and (5) absence of complete lesions of the supraspinatus tendon. The exclusion criteria were (1) rheumatoid arthritis, (2) tumours, (3) infection, (4) a history of alcohol or other substance abuse, (5) neurological disorders of the upper extremities, (6) significant cognitive impairment, (7) a diagnosis of or treatment for any psychiatric condition, and (8) failure to understand or complete the questionnaires.

### 2.2. Intervention

The US-guided percutaneous lavage and aspiration consisted of a single session, during which the subject underwent a preliminary ultrasound evaluation in the supine position on a bed, using an ultrasound scanner with a linear probe (12 MHz) set to the osteoarticular programme (Esaote Mylab 6, Esaote S.P.A, Genoa, Italy). The skin was carefully disinfected with a povidone-iodine solution, with circular centrifugal movements starting from the access point identified by the ultrasound evaluation. We then waited for the disinfectant to dry completely. The ultrasound probe, also disinfected with a chlorhexidine-based solution, was then covered with a sterile probe cover. Sterile gel was used as the transmitter. Local anaesthesia was administered using a 10 mL syringe and a 22 G needle. Under ultrasound guidance, with an in-plane approach, around 5 mL of bupivacaine 5 mg/mL was injected at the level of the subacromial bursa. Also, during the needle retraction, about 2 mL of bupivacaine was injected into the subcutaneous components.

**Figure 1:**
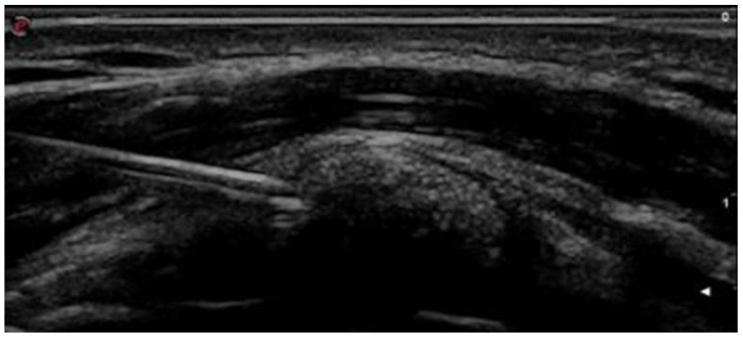
US-guided aspiration.

The approach for the calcification lavage was the one-needle technique, consisting in inserting a 16–18 G needle with a luer-lock system on a 20 mL syringe, containing 0.9% saline solution (Figure 1) in the calcific metaplasia. Again, this was performed under ultrasound guidance, with an in-plane approach. The repetitive action of pressing and releasing the plunger was repeated until the contents of the syringe became milky, at which point the syringe was replaced with a new one, always containing saline solution. This procedure was repeated until the saline solution inside the syringe was completely clear. Finally, the removal of the last 20 mL syringe was followed by inoculation at the level of the subacromial bursa, with 1 mL of methylprednisolone 40 mg/mL. The patient was then medicated and provided with an ice pack to keep in place for 15 min, during which time they remained under the observation of the medical staff. The treatment lasted a total of 30–40 min.

**Figure 2:**
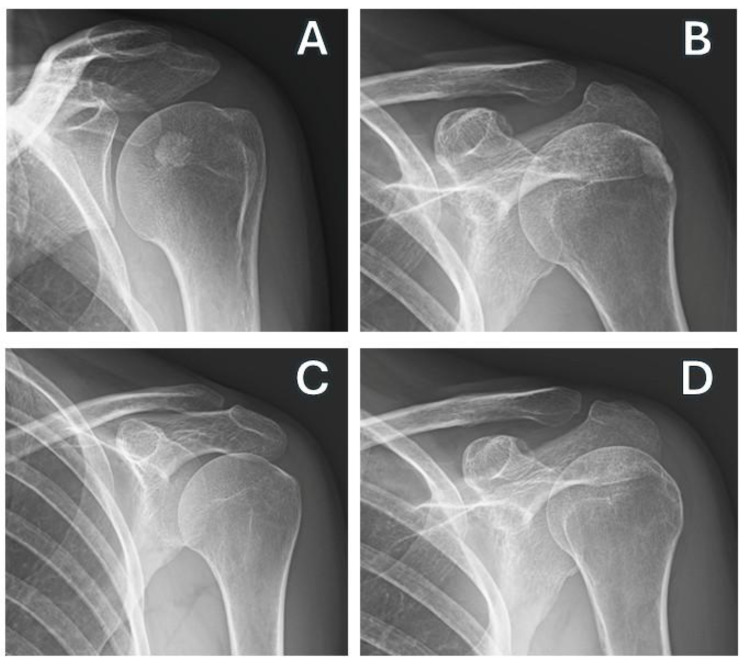
(**A**,**B**) Conventional x-rays, pre-treatment images. (**C**,**D**) Conventional x-rays, post-treatment images.

The radiologist evaluated the X-rays before the procedures and at the follow-ups (Figure 2).

The physiotherapy treatment began 7 days after the percutaneous aspiration procedure. Each session lasted around 30 min, taking place twice a week for 12 weeks. The rehabilitation was personalised to the needs of each subject, aiming to restore the shoulder joint’s complex functioning and, consequently, that of the entire upper limb. This was possible by improving the range of motion, the cuff muscle strength, and the correct scapulohumeral rhythm. Specifically, the treatment consisted of passive mobilisation and capsular stretching; assisted active exercises to increase the range of motion; and strengthening exercises with isotonic, eccentric, and plyometric contractions. The last phase, at the end of the follow-up and the 3-month point, aspired to educate the patient on home self-treatment, making them aware of the benefits of physical activity.

All interventions were performed by physiatrists and radiologists with at least 5 years of experience in musculoskeletal ultrasound. A shared protocol outlining the lavage technique, volume, and post-procedural care was applied and shared with nursing staff to ensure consistency.

### 2.3. Outcome Measures

The outcome measures analysed in the present observational study can be divided into demographic-anamnestic (such as age; sex; height; weight; body mass index; smoking habits; personal history of thyroid diseases and/or diabetes mellitus; affected side by calcific tendinopathy; the dominant side of the patient; previous treatments for the disease in question and their type; and the location of the calcification) and functional. All participants were evaluated by a physiatrist during their first outpatient visit (t0), 1 month after the procedure (t1), and at 3 months after the procedure (t2). The Numeric Rating Scale (NRS), the Gartner classification, the Disability of the Arm, Shoulder and Hand (DASH) scale, the Constant–Murley shoulder (CMS) score, and the passive range of motion (Passive ROM, PROM) of flexion and abduction were assessed. Finally, the calcification measure in centimetres was performed by a radiologist using the pre- and post-treatment radiographic images. Outcome assessments were not blinded, as the evaluators were aware of the intervention performed.

The NRS is a fast and subjective pain assessment. It is a unidimensional scale composed of 11 points (from 0 to 10), representing the severity of pain from “none” to “worst pain possible” [16]. Our primary outcome was to observe a complete or almost complete regression of pain symptoms.

The Gartner classification is widely applied to describe the radiological characteristics of calcifications [17]. Particularly, Gartner and Heyer [10] divided intratendinous shoulder calcifications into type I, dense with defined edges; type II, dense with indefinite edges; and type III, transparent with poorly distinguishable edges.

The DASH scale is a self-administered questionnaire that measures, through scores from 1 to 5 on 30 items, the level of disability related to functional activities and symptoms. The score obtained is transformed into a percentage, where 0 represents the absence of disability and 100 represents complete disability [18].

The CMS score, proposed by Constant and Murley in 1987 [19] and revised to its latest version in 2008 [20], is a functional item that estimates pain, ADLs, ROM, and the strength of the affected shoulder. The first two subjective components can have a total score of 35 points, while the last two, the objective ones, can have a total of 65 points; thus, the score varies from 0, corresponding to the worst functioning, to 100, identifying the best functionality.

Patients were monitored during the procedure and at all follow-up visits through structured clinical assessments and direct patient reports to identify any adverse events (AEs), which encompassed any peri-procedural complications; short-term symptoms (e.g., pain, swelling, and infection); and long-term sequelae (e.g., functional impairment). Furthermore, each patient was instructed to contact the study reference staff in case of AEs.

### 2.4. Statistical Analysis

The distribution of the numeric samples was assessed using the Kolmogorov-Smirnov normality test. Based on this preliminary analysis, non-parametric tests were adopted. Differences between individual variables at different time points were measured using the paired *t*-test rank test and repeated measures ANOVA. The statistical analysis was performed using Paleontological Statistics (PAST) 4.03 (Past4Project, Natural History Museum, University of Oslo, Norway). A *p*-value < 0.05 was considered statistically significant. Following the latest recommendations, for group differences (Cohen’s d or Hedges’ g), small, medium, and large effect sizes corresponded to 0.1, 0.4, and 0.8, respectively [21]. Moreover, for ANOVA, the thresholds for Partial Eta Squared are η^2^ = 0.01 (small effect), η^2^ = 0.06 (medium effect), and η^2^ = 0.14 (large effect) [22]. Participants with missing data were excluded from the analysis at the corresponding time point (complete case analysis).

## 3. Results

**Figure 3:**
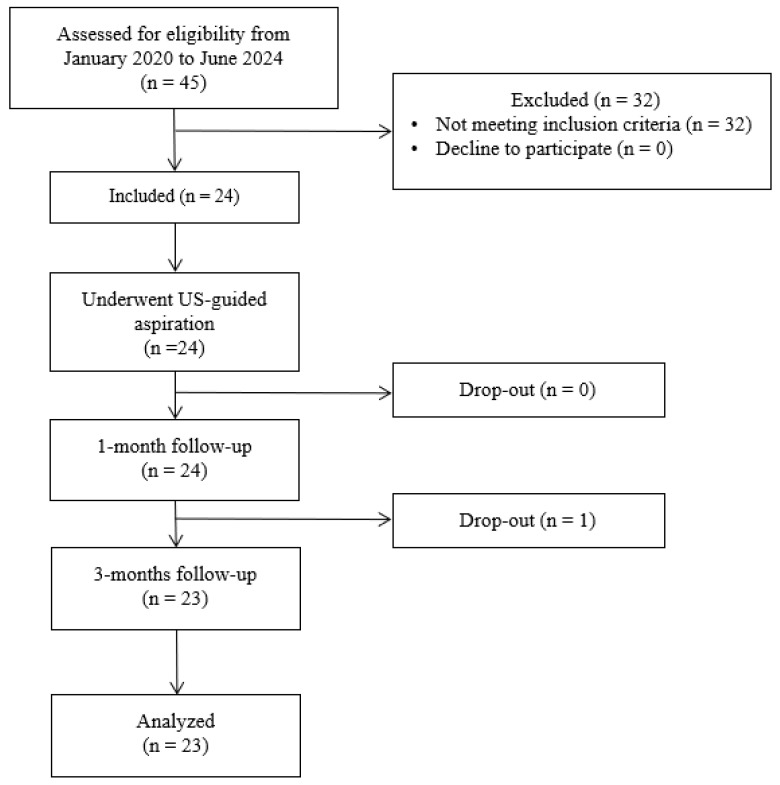
STROBE flowchart.

A total of 56 subjects affected by RCCT and who underwent US-guided percutaneous aspiration were assessed for eligibility. Of these, 32 were excluded because they did not meet the inclusion criteria. Initially, data from 24 patients were included; however, one of these patients did not participate in the planned follow-up at 3 months, consequently, the data relating to this subject were excluded from the final evaluation. The evaluation was, therefore, carried out on 23 participants, in accordance with the complete case analysis method, as depicted in the study flow chart (see Figure 3 for further details).

The study included 23 subjects affected by RCCT who had undergone ultrasound-guided percutaneous aspiration (11 male and 12 female). The median age of the patients was 58 (9). In 15 patients, the affected side was the right, corresponding to the dominant side in 75% of cases. In the remaining eight patients, the affected side was the left, corresponding to the dominant side in only 50% of the subjects. It should be noted that 21.7% of the subjects were previously treated with infiltrative approaches without benefit, with 17.3% needing another medical intervention at the end of the follow-up due to the worsening of their symptoms. Of these, one underwent treatment with external focal shock waves and three underwent intra-articular corticosteroid injections in the affected shoulder. These cases were considered as treatment failures and retained in the primary outcome analysis and classified accordingly, maintaining adherence to the intention-to-treat principles. The demographic-anamnestic data of the 23 patients are summarised in Table 1, while the functional variables are presented in Table 2.

Regarding the pain, the NRS showed a baseline score of seven (2). In total, 13% of the subjects had a Gartner classification score of three, while 34.7% had a score of one, with a median calcification measure of 1.55 (0.45). DASH and CMS showed a similar score, indicative of an intermediate level of disability. PROM was worse in 65.2% of subjects in abduction compared to flexion and was always correlated with pain. None of the participants showed peri-procedural, short-term, or long-term adverse effects.

The analysis performed on the data collected at baseline and t1 showed a statistically significant improvement in all the functional variables (*p* < 0.05). This result was particularly evident for pain, with a *p*-value of 0.001. Regarding the effect sizes, our results confirmed a statistically significant and clinically meaningful reduction in pain (NRS) one month after the procedure (*p* = 0.001, ES = 0.76), which was maintained at three months. Clinically relevant improvements were also observed in the upper limb disability score (DASH score, *p* = 0.002, ES = 0.85), the calcification diameter (*p* = 0.002, ES = 0.84), and passive shoulder flexion (*p* = 0.003, ES = 0.90). Other outcome measures—such as the Constant–Murley score, the PROM abduction, and the Gartner classification—although statistically significant, did not reach the threshold for clinical significance due to small or negative effect sizes.

Table 3 summarises the measurements at the time points and their statistical significance.

**Figure 4:**
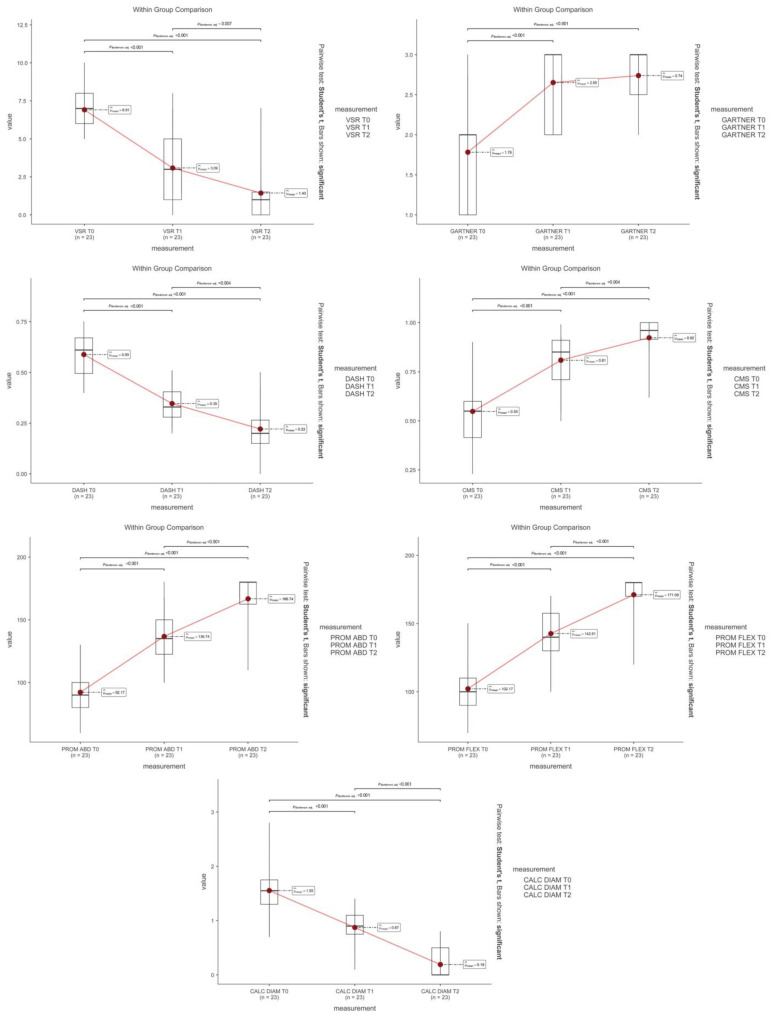
Outcome measure plot.

Moreover, the analysis performed on the data between t0 and t2 confirmed the statistically significant improvement. In contrast, no variable expressed a statistically significant modification between 1 and 3 months after the procedure (*p* > 0.05), as depicted in Figure 4.

## 4. Discussion

The present prospective case series study aimed to investigate the efficacy of ultrasound-guided percutaneous aspiration, in combination with conventional rehabilitation treatment, in pain reduction in patients with RCCT of the shoulder. The analysis of the collected data showed a significant reduction in pain one month after the procedure, a result maintained three months after treatment. Similarly, the range of motion improvement and the calcification size and consistency reduction to an overall reduction in disability, as assessed with the DASH and CMS scales.

The study focused on patients with clinically relevant calcifications (>0.8 cm) involving the supraspinatus tendon, who were likely to benefit from ultrasound-guided lavage. The exclusion criteria were applied to avoid confounding factors that could affect the clinical outcomes or the questionnaire reliability.

There is still no consensus concerning the best treatment for calcific tendinopathy of the shoulder. Ultrasound-guided percutaneous aspiration is a widely applied method in RCCT management [23], especially considering its minimal invasiveness and good tolerance by the patient. Although not free from complications, it is safe, with a low risk of peri-procedural complications, both in the short and/or long term [24].

In accordance with the International literature [25,26], 75% of our patients who underwent US-guided calcific aspiration reported a complete or almost complete regression in their pain symptoms (NRS < 2) three months after the procedure. This pain reduction was also associated with a substantial reduction in calcification: in 66% of the subjects, calcific metaplasia was no longer detectable on radiographic images. However, to date, there is still no consensus on the effectiveness of this treatment and the complete elimination of the calcific deposits in such a complex joint as the shoulder [27].

Compagnoni et al., in a 10-year-after-diagnosis radiological evaluation, identified the calcification’s persistence in 88.5% of subjects treated with several approaches (such as ESWT and US-guided percutaneous needle aspiration). Patients with smaller residual calcifications (diameter < 2 mm) showed no significant difference in terms of shoulder functionality than those with larger ones [28]. In the present observational study, the calcification measure decreased significantly after treatment and, although not statistically significant, also between t1 and t2. This suggests that the timing of intervention was probably correct in all patients, with treatment provided during the hyperacute phase. A significant reduction in pain at 1 month after treatment was observed. The benefit was maintained for the following two months, without further improvements. Four patients (17.3%) required new treatment. However, it should be underlined that these subjects presented type I calcifications at the baseline, according to Gartner’s classification, of considerable size (>1.7 cm). Also, they showed significant limitations in flexion and abduction, as well as high pain and disability in the upper limb. The characteristics of calcification (such as size, density, definition of the edges, and acoustic shadow presence or absence) correlated with the phase of formation or reabsorption, with the clinical presentation (acute or chronic pain; articular limitation), and with the potential response to treatment. Considering the size, there are no specific cut-offs, the majority of the available studies included subjects with calcifications of >0.6 cm [29,30]. Odelaar et al. [31], in a prospective cohort study, stated that smaller-sized calcification deposits represented a prognostic factor for negative outcomes. The Gartner and Heyer classification could also influence the therapy’s response. Patients with type I calcifications show less pain reduction and recovery of ROM, while type 3 calcification—smoother and softer—often correlates with acute pain and an easier aspiration [32]. Lastly, in our study, three out of the four subjects who needed another procedure following the US-guided percutaneous aspiration were female and 50% smoked. Gender, together with recreational habits such as cigarette smoking and endocrine pathologies, represented negative prognostic factors [33,34]. Moreover, the timing of the intervention is essential [24]. Calcifications that can be described as well-demarcated hyperechogenic foci, with little posterior shadow cone or with a central portion of semi-liquid consistency, are most easily treated with percutaneous aspiration [35]. Specifically, the most satisfactory results are observed in patients in the hyperalgic phase, usually corresponding to the stage of calcification formation. These subjects, moreover, require a second treatment less frequently and are less prone to developing post-procedural bursitis. This is probably due to the absence of microcrystals in the bursa [32].

Regarding the technique, in recent years, advances have allowed the use of a single needle instead of two. Several studies in the literature have demonstrated no superiority in the two-needle method in terms of pain reduction, calcification reduction, and/or ROM improvement. Generally, the two-needle technique is more suitable for harder calcifications, while the one-needle technique is more effective in the presence of soft calcifications with little acoustic shadowing [36]. This should result in a tailored approach based on the patient’s and the calcification’s characteristics. However, it is relevant to underline the methodological differences between our observational case series study and randomised controlled trials, which should lead to a cautious interpretation. Moreover, although the present study demonstrated statistically significant improvements in pain reduction and functional outcomes, it is essential to underline that p-values alone do not always reflect the clinical significance of results, which can be affected by small sample sizes. As Sullivan and Feinn [37] emphasised, including effect size measurements is critical in quantifying the magnitude of the observed effects and assessing their practical relevance. Among all of the outcomes, only pain intensity (NRS), upper limb disability (DASH), calcification size, and passive shoulder flexion demonstrated both statistical significance and medium-to-large effect sizes, thus meeting the criteria for clinical relevance. Other outcomes, despite statistical significance, showed small effect sizes and should, therefore, be interpreted with caution. These findings support a selective interpretation of results based on both statistical and clinical thresholds, especially in small-sample observational studies.

The most applied alternative option to ultrasound-guided percutaneous aspiration is ESWT. This approach is effective in eliminating calcification and in increasing function [38]. The method, however, can cause significant pain and requires multiple sessions [39]. Moreover, there are considerable differences in the EFD, the number of impulses, and the interval of administration [5]. The results obtained with US-guided percutaneous aspiration are, at least, similar to the best data published for ESWT [38]. Some authors have concluded that, even if both approaches are valid, US-guided percutaneous aspiration is associated with a greater reduction in calcification; a greater reduction in pain, with less pain during the treatment; and major cost-effectiveness.

### Study Limitations

We are aware that the results of this study should be interpreted with caution because it had several limitations.

Firstly, the small sample size and the study design did not allow a direct comparison between subjects undergoing the procedure and patients treated exclusively with conventional physiotherapy. Moreover, the impossibility of randomisation, which was not foreseen by the observational study, led to a significant selection bias, which was increased by the reduced sample size. Furthermore, the measurements at the planned follow-ups were carried out by different non-blinded physiatrists, making a measurement bias conceivable (e.g., the interpretation of subjective outcomes, such as pain). However, to reduce inter-observer variability, all the physiatrists involved received the same training on outcome assessment and followed a standardised evaluation protocol.

Secondly, the data came from patients affected by RCCT of the supraspinatus only, making it impossible to investigate the effectiveness of the method in the presence of RCCT in other locations. Another limitation was the lack of assessment of the patients’ mental status [40,41], a factor that could have influenced their treatment decisions. In addition, it has already been associated with impaired functional recovery after various procedures [42,43]. Finally, information relating to the patients’ work activities and sporting activities was collected fragmentarily and was often unavailable, not allowing for a correlation with such data.

## 5. Conclusions

Taken together, the findings of the present prospective case series study showed an improvement in the patients’ clinical and radiological outcomes after ultrasound-guided percutaneous aspiration for rotator cuff calcific tendinopathy, specifically regarding pain relief, reduction in disability, and improvements in shoulder flexion and calcification size. These results are supported by statistically significant findings and medium-to-large effect sizes. 

On the other hand, it should be considered that the patients with large type I calcifications showed a regression in their symptoms and required further treatments.

Considering the gravity of this disabling condition, which negatively impacts daily functioning, activities, and quality of life, the scientific literature should be used to further improve our knowledge of managing subjects who are affected by rotator cuff calcific tendinopathy.

Further studies with wider samples and longer follow-ups are necessary to confirm these data and to implement the use of this minimally invasive approach in clinical practice.

## Figures and Tables

**Table 1:** Characteristics of the patients involved.

Age	Sex	Height	Weight	BMI	Smoke	Dysthyroidism	Diabetes Mellitus	Affected Side	Dominant Side	Previous Treatment	Type of Treatment
59	F	1.75	80	26.1	No	No	Yes	Right	Right	Yes	Intra-articular injection
65	M	1.65	58	21.3	Yes	No	No	Right	Right	No	
58	M	1.75	71	23.1	No	No	No	Left	Right	No	
56	F	1.65	58	21.3	No	No	No	Left	Right	No	
61	F	1.58	57	22.8	No	No	No	Right	Right	Yes	ESWT
69	M	1.78	90	28.4	No	No	Yes	Right	Left	No	
51	M	1.73	68	22.7	No	Yes	No	Left	Left	No	
50	F	1.63	65	24.4	No	Yes	No	Right	Left	No	
52	M	1.76	64	20.6	No	Yes	No	Right	Right	No	
48	F	1.88	80	22.6	No	No	No	Left	Left	Yes	ESWT
54	M	1.62	61	23.2	No	Yes	No	Right	Right	No	
55	F	1.66	82	29.7	No	No	Yes	Right	Right	No	
41	F	1.58	50	20.0	Yes	Yes	No	Right	Right	No	
58	F	1.6	49	19.1	No	No	No	Right	Right	No	
80	M	1.83	75	22.4	No	Yes	Yes	Left	Right	No	
59	M	1.78	70	22.0	No	Yes	Yes	Right	Left	No	
69	M	1.86	85	24.5	Yes	No	No	Right	Right	No	
40	F	1.5	45	20.0	Yes	Yes	Yes	Right	Right	Yes	ESWT
59	F	1.65	55	20.2	No	Yes	No	Left	Right	No	
55	M	1.8	78	24.0	Yes	Yes	No	Left	Left	No	
53	M	1.85	83	24.2	No	No	Yes	Right	Left	Yes	Intra-articular injection
62	F	1.63	56	21.0	Yes	Yes	No	Left	Right	No	
69	F	1.7	60	20.7	No	No	Yes	Right	Left	No	
55.5 (7.75)		1.69 (0.10)	66.5 (19.75)	23.0 (2.57)							

In the last line, the data are presented as median (IQR). Legend: BMI—body mass index; ESWT—extracorporeal shock-wave therapy; F—female; M—male.

**Table 2:** Baseline outcomes.

NRS	Gartner	DASH	CMS	Calcification Measure (cm)	PROM Flexion (°)	PROM Abduction (°)
8	2	74	23	1.2	90	60
7	1	65	40	1.4	90	90
5	2	55	60	1.6	100	90
6	3	53	52	1.6	120	110
7	1	69	48	1.7	80	80
5	2	47	76	1.8	120	120
6	2	61	67	1.9	110	90
5	3	41	55	2.5	110	100
7	2	67	43	0.8	70	70
7	2	71	38	1.5	100	90
5	2	44	90	1.3	150	130
8	1	63	40	0.8	70	70
6	2	73	43	1.3	80	70
8	2	66	60	1.3	100	80
5	1	50	55	1.7	110	100
9	2	55	46	1.5	110	100
8	1	67	80	1.75	90	90
7	1	49	60	1.75	110	110
8	2	59	55	1.9	120	100
6	3	43	40	2.8	100	90
9	1	65	40	0.7	80	80
10	1	75	90	1.55	90	70
7	2	40	60	1.3	150	130
7 (2)	2 (1)	0.61 (0.17)	0.55 (0.18)	1.55 (0.45)	100 (20)	90 (20)

In the last line, the data are presented as median (IQR); DASH and CMS are presented as percentages. Legend: NRS—Numeric Rating Scale; DASH—Disability of the Arm, Shoulder and Hand; CMS—Constant–Murley shoulder score; PROM—passive range of motion.

**Table 3:** Intra-group variability.

Outcome Measures	T0	T1	T2	*p* Value ΔT0–T1	ES	95%LCI	95%UCI	*p* Value ΔT1–T2	ES	95%LCI	95%UCI	*p* Value RM-ANOVA	η^2^
**NRS**	2 (2)	3 (4)	1 (1.5)	0.001 *	0.76	0.44	0.99	0.54	0.72	0.25	1.17	<0.00001 *	0.57
**Gartner**	2 (1)	3 (1)	3 (0.5)	0.004 *	−0.89	−1.56	−0.80	0.157	−0.15	−0.56	0.27	<0.00001 *	0.37
**DASH**	0.61 (0.17)	0.33 (0.12)	0.2 (0.11)	0.002 *	0.85	0.16	1.52	0.44	0.94	0.44	1.43	<0.00001 *	0.64
**CMS**	0.55 (0.18)	0.85 (0.20)	0.96 (0.08)	0.003 *	−0.81	−1.36	−0.44	0.043 *	−0.76	−1.22	−0.29	<0.00001 *	0.56
**Calcification diameter (cm)**	1.55 (0.45)	0.90 (0.35)	0 (0.45)	0.002 *	0.84	0.16	1.51	0.102	0.74	0.34	1.21	<0.00001 *	0.70
**PROM flexion (°)**	100 (20)	140 (27.5)	180 (10)	0.003 *	0.90	0.45	1.66	0.108	−0.75	−1.22	−0.51	<0.00001 *	0.68
**PROM abduction (°)**	90 (20)	135 (27.5)	180 (17.5)	0.002 *	−0.96	−0.78	−1.32	0.043 *	−0.68	−1.31	−0.03	<0.00001 *	0.70

Data are presented as median (IQR). Legend: NRS—Numeric Rating Scale; DASH—Disability of the Arm, Shoulder and Hand; CMS—Constant–Murley shoulder score; PROM—passive range of motion. * = *p* < 0.05.

## Data Availability

The original contributions presented in this study are included in the article. Further inquiries can be directed to the corresponding author.

## Abbreviations

The following abbreviations are used in this manuscript:

RCCT	rotator cuff calcific tendinopathy
ESWT	focal extracorporeal shockwave therapy
STROBE	strengthening the reporting of observational studies in epidemiology
NRS	Numeric Rating Scale
DASH	Disability of Arm, Shoulder and Hand
CMS	Constant–Murley score
PROM	passive range of motion
